# A study of the use of the supraclavicular artery flap for resurfacing of head, neck, and upper torso defects

**DOI:** 10.4103/0970-0358.53005

**Published:** 2009

**Authors:** Parag Telang, Mukund Jagannathan, Maksud Devale

**Affiliations:** Department of Plastic and Reconstructive Surgery, Lokmanya Tilak Municipal General Hospital, Sion, Mumbai, India

**Keywords:** Neck defects, Island flap, Supraclavicular artery flap

## Abstract

The head and neck region is an aesthetically demanding area to resurface because of its high visibility. Tissue defects in this area often require distant flaps or free flaps to achieve an aesthetically acceptable result. The use of the Supraclavicular artery flap represents an extremely versatile and useful option for the resurfacing of head, neck and upper torso defects. Furthermore, islanding the flap gives it a wide arc of rotation and the color and texture match is superior to that of free flaps harvested from distant sites. In our study, we used the flap (both unexpanded and expanded) predominantly for resurfacing neck defects resulting from the release of post-burn contractures. However, its applicability in other indications would also be similar. Except one, all our flaps survived almost completely and the post-operative morbidity was very low. We conclude that the supraclavicular artery flap not only provides a reasonably good color and texture match but also maintains the multi-directional activity in the neck region.

## INTRODUCTION

The neck or décolleté has always been the object of pride, envy, and a lot of social discussion. The high visibility of this area poses a special challenge for the surgeon in giving an aesthetically satisfactory result. The cervical region is functionally and anatomically designed to achieve a maximum range in three-dimensional motion.

Mentosternal contractures are well-known complications after burns of any aetiology.[1] These have great physical and psychological impact on the patient. Moreover, these contractures may exert traction forces that may pull the lower lip, chin, and cheek caudally.

The various reconstructive options available for resurfacing of this region range from split thickness skin grafts and ultra-thin flaps[2,3] to local or free flaps. For the face, one has to take account of the aesthetic units and provide an appropriately thin flap to restore both form and function.[4] The color and texture match is also equally important. The commonly done procedure of release of these contractures and split-skin grafting suffers on all these counts. Again, the cervico-mental angle deserves special attention for functional and aesthetic reasons.[5,6] A loco-regional flap should also leave a minimum of donor site morbidity and preferably be hidden beneath the clothing. According to Gillies’ concept, the more adjacent the donor site is, the better the skin will match the recipient site.[7]

In our experience, the supraclavicular artery-based fasciocutaneous flap is a logical choice for head and neck resurfacing, offering advantages of good color and texture match, a relatively short operative time, and a concealed donor site. The chance of recurrence of the contracture as seen with split skin grafts is also absent.[6,8]

We decided to study the vascular territory of the flap and used tissue expansion to harvest large flaps with reliable vascularity. The use of pre-operative hand-held Doppler to identify the course of the supraclavicular vessels and pre-operative as well as intra-operative transillumination (in expanded flaps) to safeguard the vessels are, in our opinion, extremely useful measures for improving the reliability of this flap.

## MATERIAL AND METHODS

From August 2006 to September 2008, 9 supraclavicular flaps were used in 7 patients for various defects in their head and neck region. There were 2 male and 5 female patients. The indication was post-burn mentosternal contracture in 5 patients, post-burn cheek scar in1 patient, and soft-tissue sarcoma in 1 patient. The prerequisite was that at least one supraclavicular region should be uninvolved. A total of 9 supraclavicular flaps were used for resurfacing defects on the cheek (1), neck (7), and upper torso (1) regions.

Three unexpanded and 6 expanded flaps were used. The minimum flap dimension was 8cm × 6cm (horizontal × vertical; for the unexpanded) and the maximum dimension was 24cm × 14cm (for expanded flaps) [Tables 1 and 2].

**Table 1:** Patient information

Age	Sex	Donor site (Right/Left)	Flap (Natural/Expanded)
38	F	L	E
18	F	R	N
18	F	L	E
45	M	L	N
20	F	R + L	E × 2
36	F	R	N
35	M	R + L	E × 2
Total	M:2,F:5	R:4,L:5	E:6,N:3

**Table 2:** Flap-related details

Surgical indication	Flap dimensions (cm)	Arc of rotation (degrees)	Survival	Donor site closure
PBC	18 × 16	150	Complete	P
PBC	8 × 6	180	2 cm distal necrosis	P
PBC	10 × 6	120	Complete necrosis	P
PBC	12 × 8	150	Complete	P
PBC	16 × 14 and 14 × 7	180 each	Complete	P
Soft-tissue sarcoma	8 × 8	150	Complete	P
PBC	14 × 7 and 16 × 14	180 each	3 cm distal necrosis of Rt flap	P

PBC = post-burns contracture; P = primary closure

The flap was islanded on its fascial pedicle in all cases and all donor sites could be closed primarily after undermining. All the patients were counseled pre-operatively about a visible scar over the donor site and the possibility of stretching of the scar post-operatively was explained.

The procedure was performed under general anesthesia with endotracheal intubation.

### Flap design

The flap marking was done conforming to the territory over the shoulder cap as described by Pallua *et al.*[6] A hand held Doppler was used to localize the supraclavicular vessel as it crossed the clavicle and its further course and direction was mapped [Figure 1a]. If the neck was unburnt, the sternocleidomastoid, external jugular vein, and clavicle were marked to obtain the exit point of the vessel. The flap territory was marked out on both sides of the ascertained course of the vessel. In cases where a tissue-expander (TE) was to be inserted, the anterior incision was taken and the expander was inserted in an immediate supramuscular plane.

**Figure 1a:**
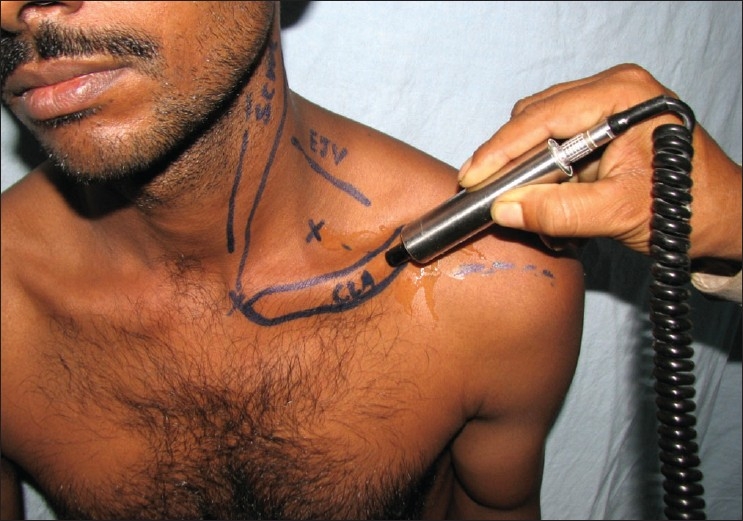
Use of hand- held doppler to mark the course of the supraclavicular vessels pre-operatively

The final flap design and dimensions were always made after the defect was created. In expanded flaps, after localization of the vessel using transillumination, the flap was designed to be centered on the vascular axis. The surgery began with the release of the contracture. The defect was created after release of the contracture and the flap size was tailored accordingly.

### Flap elevation

The flap elevation was standardized by starting the elevation from the lateral (distal) aspect and progressing medially in a subfascial plane. The communicating perforators from the deltoid branch of the thoraco-acromial axis and posterior circumflex humeral artery were sacrificed. If possible, incisions medial to the clavicle were avoided to prevent visible scars. Near the point of exit of the supraclavicular vessels, the dissection was done preserving a fascial pedicle of about 3cm in width. This is the pivot point for the flap [Figure 1b]. The length of the subcutaneous pedicle depended on the edge of the defect medial to the clavicle. The raised flaps were observed for bleeding from the distal end to ensure flap viability. In cases where the scarring reached up to the clavicle, the flap inset started immediately from the medial edge of the clavicle. All the flaps were islanded.

**Figure 1b:**
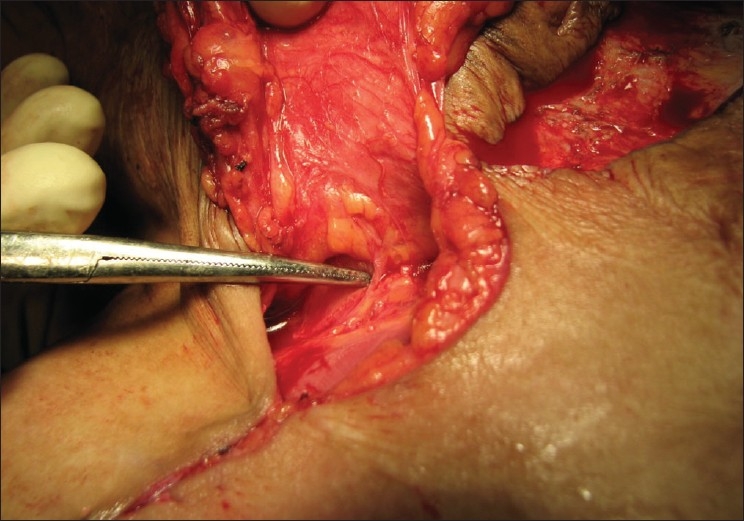
Close-up image of the supraclavicular vessels seen at the exit point

### Flap inset

After the defect was created, the flap was tunneled below the cervical incision along an arc of 120-180 degrees. The inset was done in two layers: Subcutaneous with absorbable 3-0 Polyglactin 910 (Vicryl^®^) and skin with non absorbable 5-0 Nylon (Ethilon^®^) sutures. A suction drain was used to drain the flap and the donor site, which was removed by the third post operative day. All donor sites could be closed with undermining. A simple gauze dressing was applied and held in place with adhesive paper tapes. No splintage was used immediately post-op. Our average operative time was 2-3 hours, similar to what was reported by Rashid *et al*.[9]

The patient was nursed with a pillow under the shoulder blades to maintain the neck in extension. A commercially available soft Philadelphia Collar^®^ was applied after the third post-operative day and the patient was discharged. The first follow-up visit was on the fifth post-op day for change of dressing and examination of the flap. The sutures were removed on the 10^th^ post-op day at the second follow-up visit. The patients were followed-up at 1, 3, and 6 months post-op and then at yearly intervals.

## CASE REPORTS

### Case 1

A 38-year-old female with a cervicomental contracture on the left side of the neck [Figure 2a] was given the option of release with split skin grafting or expansion of the left supraclavicular area. After due considerations she opted for the latter. A 15cm × 6cm × 5.5cm rectangular 500 cc tissue expander was placed beneath the left supraclavicular area and expanded to 750 cc. An expanded flap measuring 18cm × 15cm was used to resurface the final defect [Figure 2b].

**Figure 2a:**
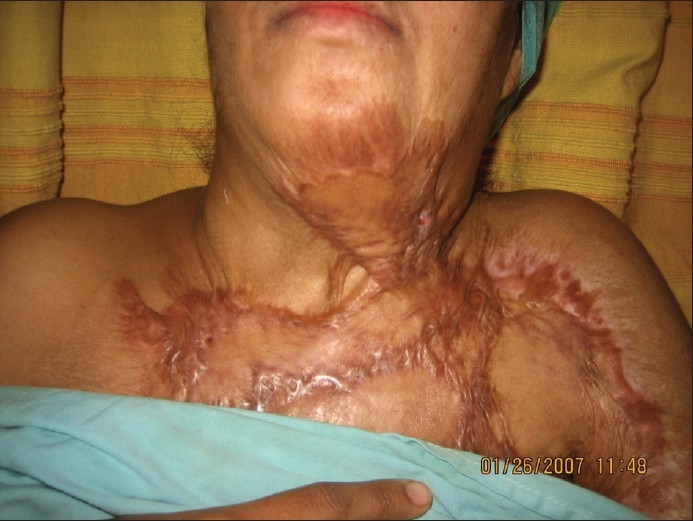
Case 1: Pre-operative appearance showing post-burns contracture band on lateral aspect of neck with restriction of neck extension

**Figure 2b:**
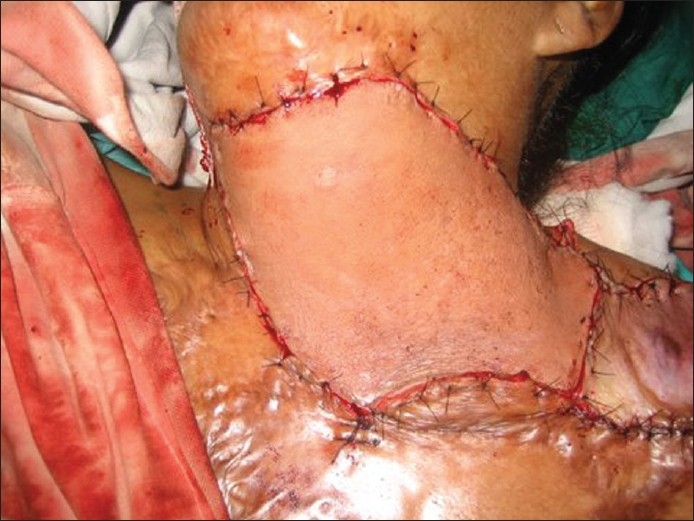
Intra-operative image showing the flap after inset in the defect

Her post-operative recovery was uneventful. She later developed a contracture band at the medial inset that was not part of the original contracture but had developed as a result of straight line closure. This was released with Z-plasty under local anesthesia. At the 20-month follow-up visit, this patient had good color, texture match, and neck extension of more than 150 degrees [Figures 2c and d].

**Figure 2:**
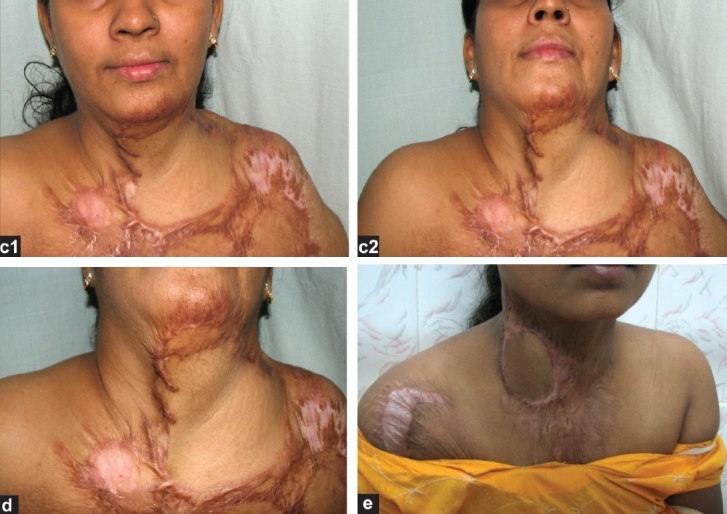
(c1,2) and (d): Post-operative appearance of the same patient at 4 weeks post-op showing a well-settled flap with good colour match and complete neck extension. Flap of 18 cm × 15 cm dimensions was elevated. (e) Case 3: Post-operative appearance with the flap advanced to cover the distal 2-3 centimeter that was lost

### Case 2

An 18 year-old female presented with a post-burn scar over her left cheek. We expanded her left supraclavicular area with a cylindrical expander to resurface the cheek scar. Though the expander migrated beyond the shoulder cap region in the post-insertion period, we went ahead with the surgery. A flap measuring 10cm × 6cm was harvested. The flap did not bleed well on the table and showed ischemic changes immediately after the operation. We lost the flap in its entirety. The resulting defect was later covered with a split-thickness skin graft.

### Case 3

A 19-year-old female with a post-burn scar over the right lateral side of the neck underwent resurfacing using a right supraclavicular flap measuring 8cm × 6cm. The distal 1–2cm of the flap necrosed but we could advance the rest of the flap to cover the defect [Figure 2e]; however, the result was suboptimal aesthetically.

### Case 4

A 45-year-old male with severe torticollis of the neck due to post-burn contracture on the left side of the neck underwent contracture release and resurfacing of the defect using a flap about 12cm × 8cm. Not only was the entire contracture released completely, the color and texture match provided was quite good [Figure 2f]. The donor site was closed primarily. At the 18-month follow-up visit, the flap had maintained its aesthetic and functional qualities and the patient was very happy [Figures 2g and 2h].

**Figure 2:**
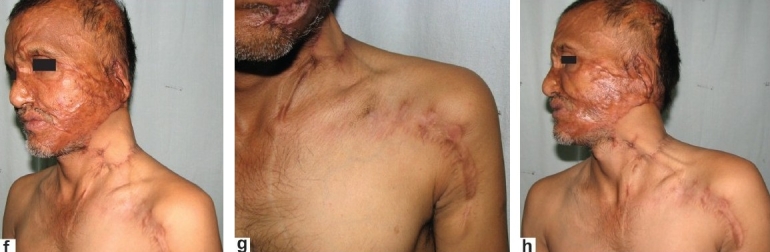
(f,g,h) Case 4: Post-operative appearance at 6 weeks showing completely well-settled flap with complete release of the contracture. The second image shows the well-healed donor site

**Figure 3b:**
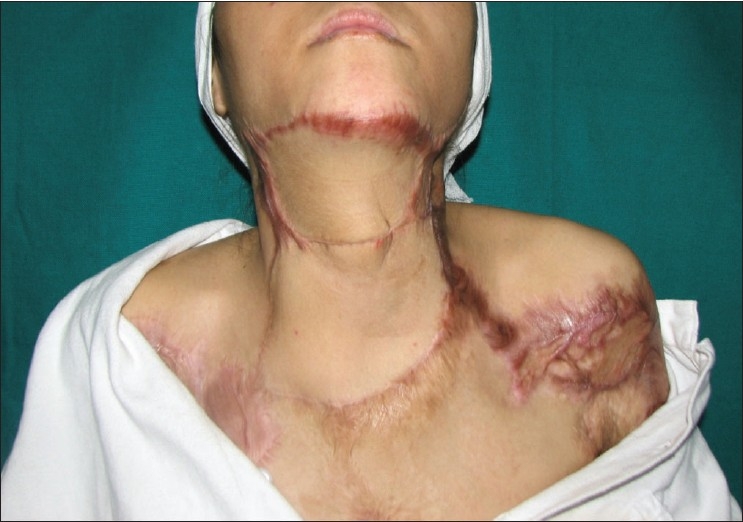
Post-operative appearance at 12 weeks post-op showing complete excision of the scar and both flaps well-settled

**Figure 5(b):**
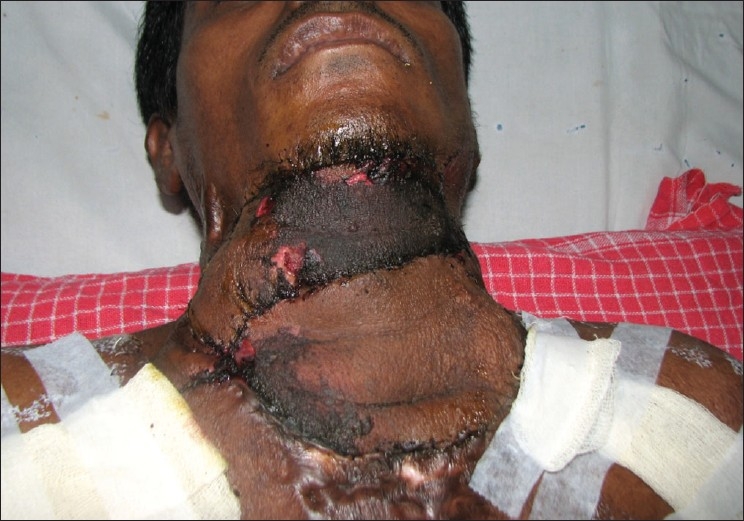
Early post-operative image showing superficial necrosis of the right flap which required debridement and split-thickness skin grafting

### Case 5

A 22-year-old fair female with severe scarring over the entire mento-sternal region underwent tissue expansion of both supraclavicular territories using curved cylindrical expanders measuring 12.5cm × 5.5cm × 6cm (vol 350cc each) [Figure 3a]. After excision of the defect, two flaps, the right flap measuring 16cm × 14cm and the left flap measuring 14cm × 7cm, were harvested and the defect resurfaced with the left flap placed cranially and the right one placed caudally. The recovery was uneventful and the color and texture match as well as the pliability of the flaps was satisfactory [Figures 3c and 3d]. At the 8-month follow-up visit, the neck extension was complete and the cevico-mental definition was maintained. The donor sites were closed primarily.

**Figure 3a:**
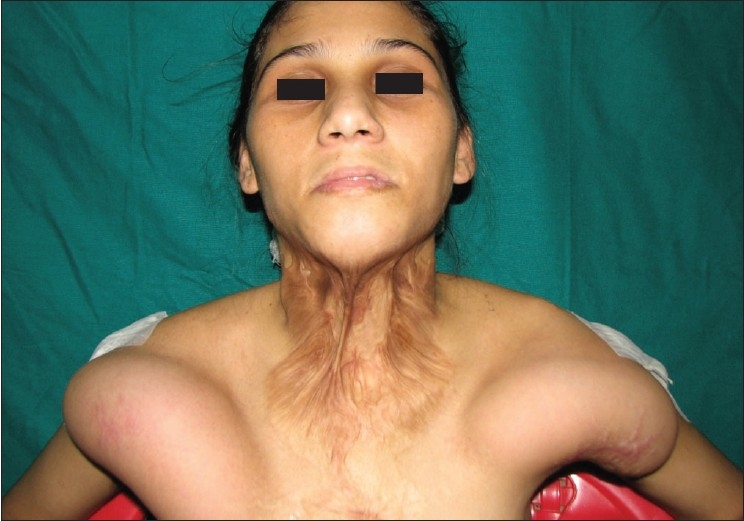
Case 5: Pre-operative appearance with bilateral tissue-expanders *in situ*

**Figure 3c:**
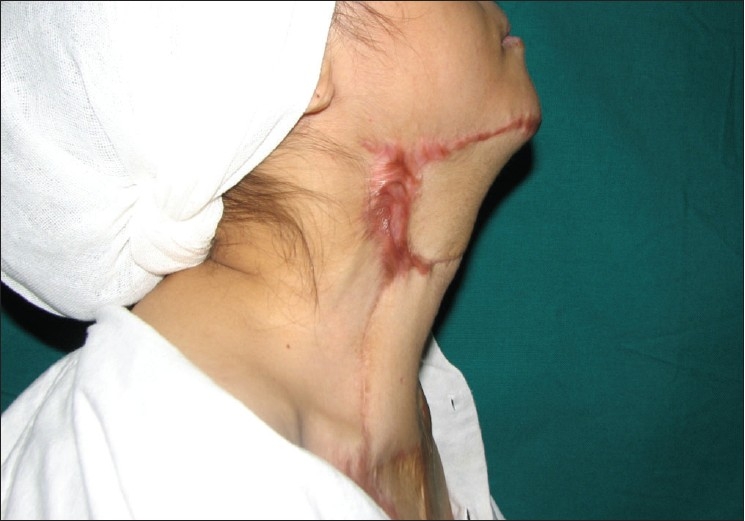
Appearance of the same patient from lateral aspect with complete neck extension

### Case 6

A 38-year-old female presented to us with a soft-tissue tumour of the right infraclavicular region that measured 6cm × 6cm [Figure 4a]. There was no involvement of the underlying muscles. We resurfaced this defect using a supraclavicular flap measuring 8cm × 8cm and closed the donor defect primarily [Figure 4b]. At the 3-month follow-up visit, the flap was completely settled and had blended well with the surrounding skin [Figures 4c and 4d].

**Figure 4a:**
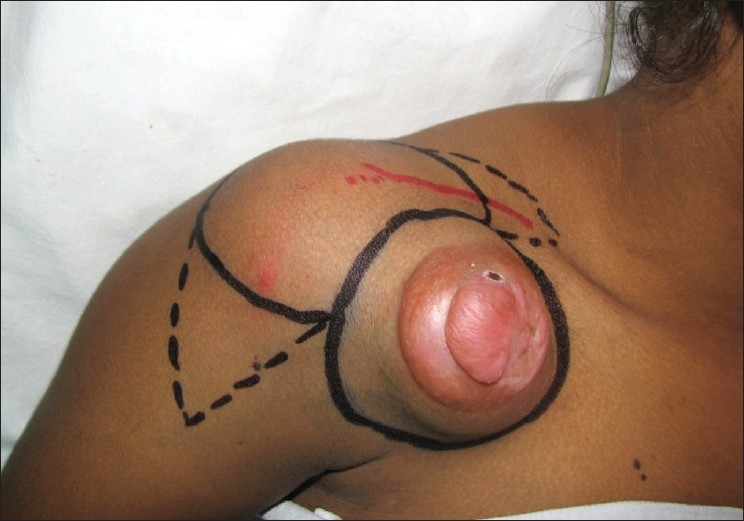
Case 6: Pre-operative appearance with the supraclavicular flap marked along with the vessel

**Figure 4b:**
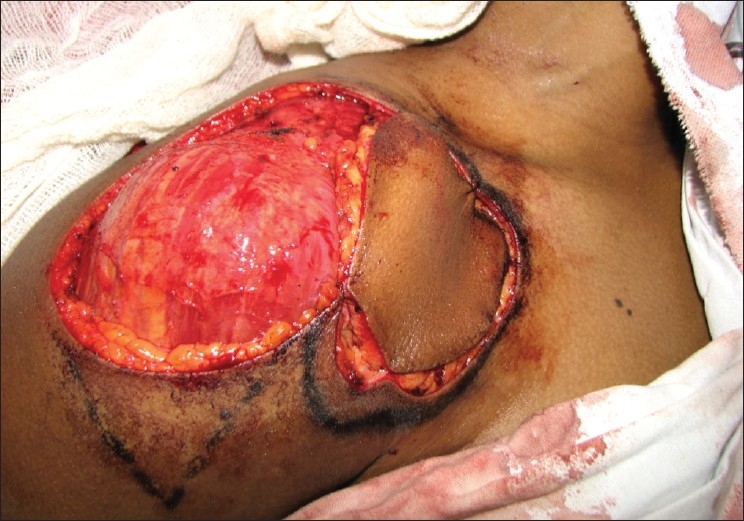
Intra-operative image with the flap being inset into the defect

**Figure 4(c,d):**
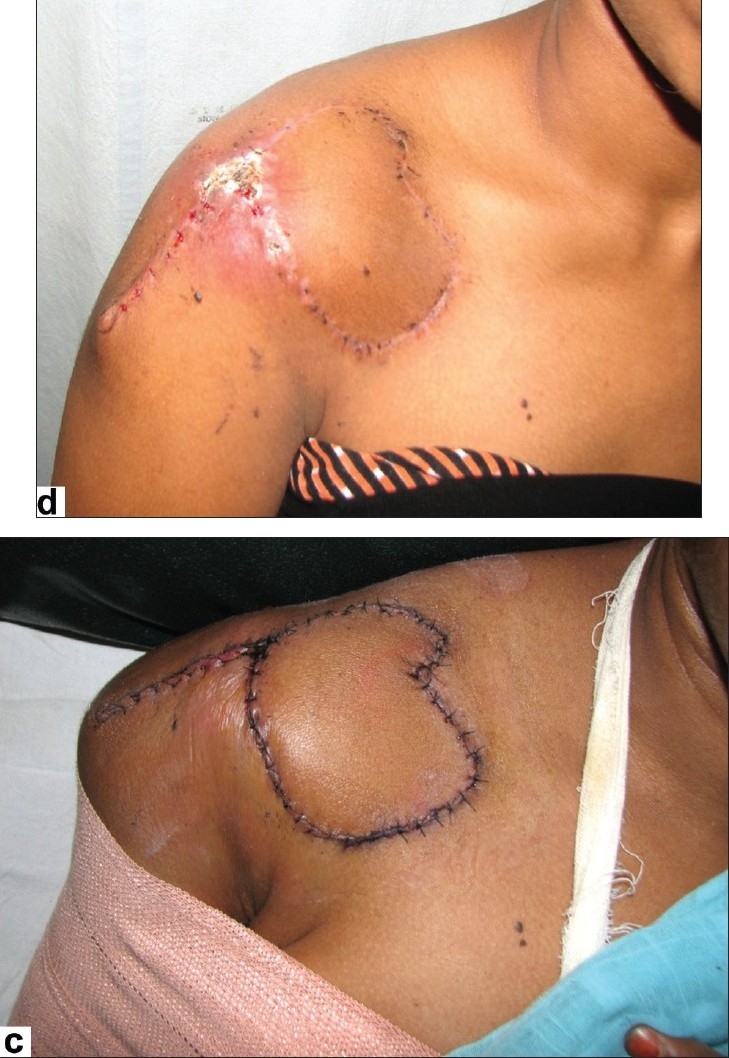
Appearance at 6 days and 6 weeks post-op with healed donor site showing minimal stretching of the scar

### Case 7

A 35-year-old male patient with severe keloidal scarring of the cervico-mental region was our most recent patient. We placed two rectangular expanders on both sides [Figure 5a]. After excision of the scar, supraclavicular flaps were raised on both sides. The right flap measured 14cm × 7cm and the left flap measured 16cm × 14cm. Using the standard procedure, the defect was resurfaced with the right flap positioned cranially and the left flap placed caudally. The distal 2-3cm of the upper (right) flap necrosed [Figure 5b] but could be covered with a split skin graft. The cervico-mental definition was achieved and neck extension up to 150 degrees was seen [Figure 5c].

**Figure 5(a):**
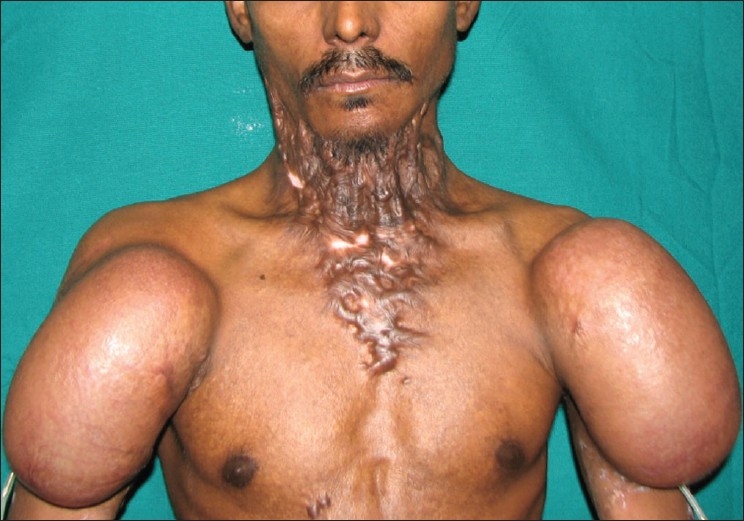
Case 7: Pre-operative appearance and course of the vessels marked on right side

**Figure 5(c):**
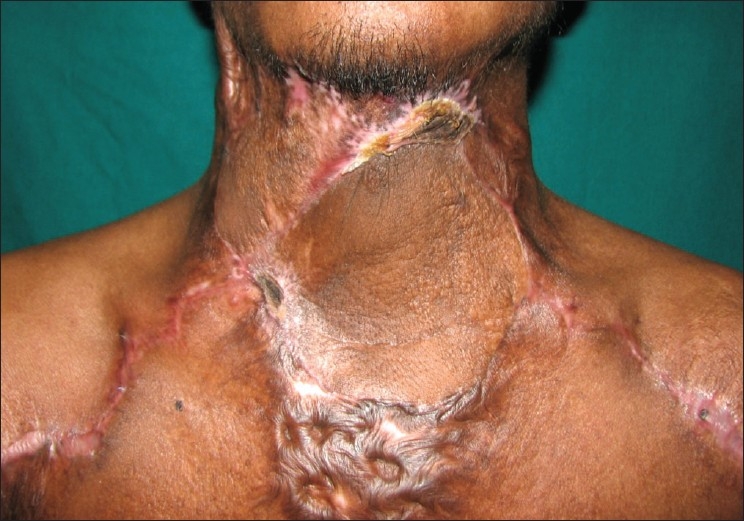
Follow-up image at 12 weeks post-op with well-healed flaps, donor sites and complete neck extension achieved

## RESULTS

All flaps except for one did well. The fact that the expander migrated beyond the known described territory of the flap might have been the cause of this failure. Distal flap necrosis of 2 to 3cm in 2 patients were avoidable with better flap planning, which we practiced in our later cases.

Our patients were mostly happy with their final appearance. With donor sites closed primarily and the scar hidden underneath clothing, we found it easier to convince subsequent patients to opt for the procedure. We observed stretching of the donor site in 3 patients but there was no hypertrophy or keloidal change (Even in our latest patient with known keloidal tendency, at 3 month follow-up).

Elaborate neck splintage and bulky dressings generally used for split skin grafts were not needed and the average time from post-operative recovery to resuming work was quite short, about 8 to 10 days.

The patients were seen at 1-, 3-, and 6-month intervals. Adjuncts such as silicone gel sheet application and pressure garments were used if needed.

Z-plasties were done to release minor areas of tightness due to straight-line scars and not for correction of failures of the release process. We were sometimes hesitant to use primary z-plasties due to concerns about the vascularity of the flap at the primary surgery; hence, they were done secondarily.

The clinical appearance of all flaps resembled the natural contouring of the cervical silhouette. The color and texture match was maintained and none of the patients had any recontracture.

## DISCUSSION

The expected esthetics of the head and neck region region in terms of the color, texture match, and the functional dexterity that is desired makes any reconstruction in this region a very challenging one. Though the release of contractures and split-skin grafting is a readily available option, it performs poorly on many counts. The lack of color and texture match, and chance of re-contracture leads to dissatisfaction amongst patients. At the other end of the spectrum, options like ultra-thin free flaps need specialized equipment and long operative time.

The flap based on the supraclavicular branch of the tranverse cervical artery has had a long and chequered history. It was first described by Kazanjian and Converse as “in charretera” or acromial flap.[10] The Demergasso flap,[11] which was described by Mathes and Nahai,[12–14] and several other flaps have evolved into the supraclavicular artery flap as we know it today. Cormack and Lamberty defined the flap as a laterally extended cervico-humeral flap and published an article about its vascular anatomy in 1983.[15,16] The first anatomical studies of the cervicohumeral flap were performed in 1977 by Mathes and Vasconez.[17,18]

The vessel described by them as “an ascending branch of the artery cephalad to the clavicular insertion of the trapezius muscle” was named the supraclavicular artery by Lamberty.

The supraclavicular fasciocutaneous island flap was actually introduced by Lamberty in 1979.[19] He correctly described the supraclavicular artery as a perforator that arises from the transverse cervical artery in 93% of cases or from the suprascapular artery in 7% of cases.[20]

The flap fell into obscurity until 1997 when further fundamental studies were carried out by Pallua *et al.*[21] who described the supraclavicular island flap for releasing postburn mentosternal contractures as a reliable and useful flap. Three years later, Pallua and Noah[22] further defined the anatomical features of the supraclavicular artery by their studies on cadavers.

According to Cormack and Lamberty,[23] an anatomical territory including the main blood flow into a flap is linked to the next anatomical territory through choke vessels and these two anatomical territories, including choke vessels, are the basic flap survival area. Keeping this guiding principle in mind, in our study we found that the supraclavicular flap can be safely elevated within dimensions of 20cm × 10cm. Use of tissue expansion greatly amplifies the total area available. Furthermore, the use of flaps from both sides not only greatly improves the total area that can be resurfaced but also eliminates the tension on the suture line while attempting to cover distal-most portions of the neck and head.

However, it must be taken into account that the distal portion of the flap may be perfused in a retrograde fashion by branches of the posterior circumflex humeral artery through the anastomoses over the point of the shoulder, hence the role for delay in unexpanded flaps remains.

In our experience, the use of the supraclavicular flap both with and without expansion is an excellent option for resurfacing of head and neck defects. The flap scored well in all parameters studied including reliability, reach, and quality of resurfacing [Tables 1 and 2]. The overall patient satisfaction was also good.

Anatomical aberrations in the course of the vessel should be kept in mind while harvesting and islanding it. The use of a pre-operative hand-held Doppler and transillumination (in expanded flaps) to mark the course of the vessel are two adjuncts that are worth emphasizing. However, in extensive burns involving the head and neck region, the supraclavicular area is also involved in many cases and hence may not be available for resurfacing using this flap. The other technical points to be reiterated are preservation of a fascial pedicle of 3–4cm near the point of exit of the vessel that greatly improves the reliability of the flap. We think that one should stop short of the clavicle in order to prevent a scar in the visible aspect of the neck. We also noticed that elevating the flap in a subfascial plane ensures that the vessel is always included in the flap. It's a good idea to include a small portion of the platysma at the pivot point to further safeguard the vessel. The incidence of distal necrosis that we encountered (2 out of 8) compares with that described in the literature.[16,24] We have had only one failure so far and we attribute it to our learning curve of combining tissue expansion with this flap.

## CONCLUSIONS

Based on our experience so far with the use of a natural and expanded supraclavicular artery flap for head and neck resurfacing, we can safely conclude that this is an excellent reconstructive option in the armamentarium of a plastic surgeon to be offered to the cosmetically conscious patient. We have no hesitation in recommending the more widespread use of this flap and future possible use as a free flap based on transverse cervical artery is already on the anvil.
